# Estimating clinical and economic burden of pneumococcal meningitis in Malaysia using Casemix data

**DOI:** 10.1186/1472-6963-12-S1-O4

**Published:** 2012-11-21

**Authors:** Nimetcan Mehmet Orhun, A Zafar, M Amrizal, Md Isa Zaleha, S Saperi, Syed Aljunid

**Affiliations:** 1 United Nations University International Institute for Global Health, Kuala Lumpur, Malaysia; 2 Department of Community Health, UKM Medical Center, Kuala Lumpur, Malaysia; 3 International Centre for Case-Mix and Clinical Coding, UKM Medical Centre, Kuala Lumpur, Malaysia

**Keywords:** Public Hospital, Economic Burden, Hospital Cost, Annual Cost, Pediatric Case

## Background

Pneumococcal disease kills over 1.6 million people each year. The vast majority of its victims come from developing countries. Meningitis cases due to *S. pneumoniae* have one of the highest mortality rates in pneumococcal diseases. However there is no data on clinical and economic burden of pneumococcal meningitis (PM) in Malaysia. Aim of this study was to estimates the clinical and economic burden of PM in Malaysia

## Methodology

The clinical and economic burden of pneumococcal meningitis (ICD code G00.1) was assessed using a two years retrospective review of patient’s medical records (2008-2009) from four public hospitals in Malaysia. The cases of PM were identified the using ICD 10 diagnoses codes and were assigned CBG group using MY DRG from UNU Casemix system. Costs of PM were estimated based on the MY DRG using step down costing methodology from hospital cost data. The annual cost of the disease was stratified for pediatric and adult cases.

## Result

There are 2,809 PM cases annually; out of these 1,392 belongs to the pediatric age group while 1,417 were adult cases. Cost per episode of pneumococcal meningitis was calculated. The cost for inpatient pediatric pneumococcal meningitis was estimated as RM 6,027 while for inpatient adult case was estimated as RM 4,985. The cost for pediatric outpatient pneumococcal meningitis visit was RM 824 while that for adult case was RM 515. Total direct cost for pneumococcal meningitis is estimated to be RM 3,737,584, of which 52% (RM 1,785,811) were due to pediatric cases while 48% (RM 1,951,773) were generated by adult cases in Malaysia.

## Conclusion

PM is presented a significant burden for Malaysian population and society. The disease burden can be reduced through vaccination.

